# Comment on Muzzioli et al. Are Front-of-Pack Labels a Health Policy Tool? *Nutrients* 2022, *14*, 771

**DOI:** 10.3390/nu14102165

**Published:** 2022-05-23

**Authors:** Hassan Aguenaou, Nancy Babio, Mélanie Deschasaux-Tanguy, Pilar Galan, Serge Hercberg, Chantal Julia, Alexandra Jones, Georgios Karpetas, Bridget Kelly, Emmanuelle Kesse-Guyot, Lamprini Kontopoulou, Marie-Eve Labonté, Cliona Ni Mhurchu, Igor Pravst, Simone Pettigrew, Elio Riboli, Jordi Salas-Salvadó, Bernard Srour, Mathilde Touvier, Stefanie Vandevijvere

**Affiliations:** 1 Joint Research Unit in Nutrition and Food, RDC-Nutrition AFRA/IAEA, Ibn Tofail University-CNESTEN, Rabat, Kenitra 14000, Morocco; aguenaou.hassan@uit.ac.ma; 2 Departament de Bioquimica i Biotecnologia, Unitat de Nutrició Humana. (IISPV), Universitat Rovira i Virgili, 43201 Reus, Spain; nancy.babio@urv.cat (N.B.); jordi.salas@urv.cat (J.S.-S.); 3 Consorcio CIBER, M.P. Fisiopatología de la Obesidad y Nutrición (CIBERObn), Instituto de Salud Carlos III (ISCIII), 28014 Madrid, Spain; 4 Sorbonne Paris Nord University, Inserm U1153, Inrae U1125, Cnam, Nutritional Epidemiology Research Team (EREN), Epidemiology and Statistics Research Center—University of Paris (CRESS), 93017 Bobigny, France; m.deschasaux@eren.smbh.univ-paris13.fr (M.D.-T.); p.galan@eren.smbh.univ-paris13.fr (P.G.); s.hercberg@eren.smbh.univ-paris13.fr (S.H.); e.kesse@eren.smbh.univ-paris13.fr (E.K.-G.); b.srour@eren.smbh.univ-paris13.fr (B.S.); m.touvier@eren.smbh.univ-paris13.fr (M.T.); 5 Public Health Department, Avicenne Hospital, Assistance Publique des Hôpitaux de Paris (AP-HP), 93000 Bobigny, France; 6 The George Institute for Global Health, University of New South Wales, Sydney, NSW 2052, Australia; ajones@georgeinstitute.org.au (A.J.); spettigrew@georgeinstitute.org.au (S.P.); 7 Laboratory Teaching Staff, Faculty of Medicine, School of Health Sciences, University of Thessaly, 41500 Larissa, Greece; gkarpetas@uth.gr; 8 Early Start, School of Health & Society, University of Wollongong, Wollongong, NSW 2522, Australia; bkelly@uow.edu.au; 9 Laboratory Teaching Staff, Nursing Department, University of Thessaly, 41500 Larissa, Greece; lamprini2@yahoo.gr; 10 Centre Nutrition, Santé et Société (NUTRISS), Institute of Nutrition and Functional Foods (INAF), Université Laval, Québec City, QC G1V 0A6, Canada; marie-eve.labonte@fsaa.ulaval.ca; 11 School of Nutrition, Université Laval, Québec City, QC G1V 0A6, Canada; 12 National Institute for Health Innovation, The University of Auckland, Auckland 1142, New Zealand; c.nimhurchu@auckland.ac.nz; 13 Nutrition and Public Health Research Group, Nutrition Institute, Trzaska Cesta 40, 1000 Ljubljana, Slovenia; igor.pravst@nutris.org; 14 Department of Epidemiology and Biostatistics, Imperial College London, London W2 1PG, UK; e.riboli@imperial.ac.uk; 15 Department of Epidemiology and Public Health, Service of Lifestyle and Chronic Diseases, 1050 Brussels, Belgium; stefanie.vandevijvere@sciensano.be

As scientists working and publishing in the field of front-of-pack nutrition labelling (FOPNL) for many years, we have read with interest and concern the narrative review regarding their effectiveness by Muzzioli et al. [1].

First, the authors appear to consider that the premises underlying FOPNL—to inform consumers on the nutritional composition of foods from a health perspective and orient them towards healthier purchases—are not the object of consensus in the scientific community, despite them being clearly stated by the WHO [2]. In addition, they appear to place purely informative labels (such as the NutrInform Battery) and interpretive labels (Nutri-Score, Warning labels, Multiple Traffic Light) at the same level, while there is a clear scientific consensus that interpretive front-of-pack labels are more effective and equitable, and should be promoted (WHO principle n°7) [2]. Finally, FOPNL have been shown to act as drivers for reformulation, improving the overall nutritional environment [3].

Second, while FOPNL research usually relies on a clear theoretical framework [4], and grades the evidence provided by each type of study, the review conflates results from experimental studies, consumer surveys, cohort studies, and even studies not conducted with consumers at all to present a perspective that lacks a clear objective.

Finally, multiple studies investigating the performance of labels, including a very important network meta-analysis [5] and studies conducted in multiple countries comparing the effects of various types of labels, were entirely omitted from the review [6,7,8,9,10,11,12,13,14,15,16,17,18,19,20,21,22]. These studies provide outstanding information as to the comparative effects of FOPNLs to help consumers identify healthier foods and make healthier purchases, and contribute to reduce the burden of nutrition-related disease [23,24]. Of great concern is that when studies on performance are referenced, they are misrepresented, instead reporting the results of preference elements within those same studies. For example, the study by Hagmann et al., which found a clear higher performance of Nutri-Score over all other labels, is relayed as finding that ‘most of the participants […] considered the Nutri-Score the least useful’ [25], which clearly misrepresents the results of the study.

These elements lead to an overall biased and misleading view of the literature, diminishing the value of multiple studies consistently showing that interpretive labels (Nutri-Score and Warning labels, in particular) lead to healthier food choices, as well as overstating the merits of non-interpretive labels, which have consistently been shown to be unable to produce significant modifications in dietary choices.

That FOPNL are only one of the many policies necessary to tackle the obesity epidemic has never been in doubt. However, it is also clear that FOPNLs are a useful health policy tool and, more importantly, *interpretive labels* such as the Nutri-Score constitute an evidence-based health policy tool [26], as reflected in the multiple studies that were apparently overlooked or misrepresented in this study.
